# Using former carers’ expertise in peer support for carers of people with Parkinson’s Disease

**DOI:** 10.1038/s41531-022-00381-0

**Published:** 2022-10-15

**Authors:** Angelika D. Geerlings, Marjan J. Meinders, Bastiaan R. Bloem, Marjolein A. van der Marck

**Affiliations:** 1 Radboud university medical center; Donders Institute for Brain, Cognition and Behaviour; Department of Neurology; Center of Expertise for Parkinson & Movement Disorders, Nijmegen, The Netherlands; 2 Radboud university medical center, Radboud Institute for Health Sciences, Scientific Center for Quality of Healthcare, Nijmegen, The Netherlands; 3 Radboud university medical center, Radboud Institute for Health Sciences, Department of Geriatrics, Nijmegen, The Netherlands

**Keywords:** Health care, Public health, Quality of life

## Abstract

Informal carers gain unique experience and knowledge when caring for a loved person. However, this knowledge often remains unused after their loved one with Parkinson’s disease (PD) has passed away. Hence, two opportunities are currently being missed: sharing this unique experience could support current informal carers and offer the bereaved former carers the option to continue to fulfil a meaningful role. This study aimed to identify the unmet needs of current carers, and to examine the interest, willingness and requirements of both current and former carers for peer-to-peer support. Data were collected from August 2020 to February 2021 through questionnaires examining (1) resources and needs for support; (2) topics for support and advice; and (3) preferences for peer-to-peer initiatives. Open questions were analyzed thematically, after open coding. In total, 141 current and 15 former informal carers participated. Current carers were mainly women (68%) and partner of a person with PD (86%). Former carers were mainly women (80%) who had cared for a partner or parent (53%; 47%) with PD. Almost half of the current carers expressed need for additional support in finding balance, changing relationships, and learning how to cope with lack of emotions and motivation. Half of the carers were positive about the opportunity to exchange experiences and knowledge with former carers. Willingness among former carers for providing peer-to-peer support was high (87%). In both groups, having a degree of commonality with peers was considered an essential requirement. These findings provide guidance for developing peer-to-peer support programs, incorporating former carers.

## Introduction

Parkinson’s disease (PD) is an unpredictable, chronic and progressive disease, characterized by an individually determined spectrum of motor and non-motor symptoms^1^. PD has a great impact on the quality of life and health outcomes, both for individuals affected by the disease and for those taking care of them, typically spouses or children^2^. When the disease starts to impact people’s physical functioning and autonomy, informal carers often step in to support activities of daily living^3,4^. Non-motor symptoms, such as vivid sleeping or hallucinations, not only disrupt a patient’s sleep, but often also impact the sleep quality of the informal carers^3–6^. Informal carers for persons with PD has been linked to a number of detrimental effects for the carers, including poor health outcomes, psychological stress and strain, depressive symptoms and reduced sense of wellbeing^7–10^. These physical and psychological problems experienced by those caring for a person are referred to as carers burden^11^. Previous research identified mental stress – constant worrying about the person with PD, including their safety – and loss of the relationship with the person with PD, as the factors contributing most to experienced carers burden^12,13^. As the disease progresses, the dependency of people with PD on assistance from informal carers increases and, consequently, further impacts the carers burden and strain negatively^14^.

To alleviate carers burden, access to supportive networks that provide information, guidance and practical advice on chronic disease and care management has been reported consistently in the literature as being possibly beneficial^15^. Indeed, previous studies have shown that informal carers of people with PD often report feeling on their own in finding a way to cope with the uncertainties of the disease and with the many everyday challenges, while they receive little support and understanding for their situation from their social environment^4,16,17^. In response, these studies have suggested that support programs are needed that are tailored to the unique needs of informal carers. Peer support – i.e., support that is delivered by persons who have faced a similar situation – has been suggested as a possibility to support carers^18,19^. In addition, a large-scale study among people with PD showed the negative impact of social isolation due to the COVID-19 pandemic on quality of life with PD^20^. Social isolations comes along with feelings of loneliness and decreased social interactions, and is also reported among carers of people with PD^21,22^. Providing care for a person lead to decreased independent and freedom and can bring couples emotionally further apart from each other^23^. Previous studies found that carers attending support groups report less loneliness and more perceived social support^21^. However, very limited previous research showed that such peer support offers various benefits, including providing access to information and advice, increasing levels of self-esteem and confidence, and receiving empathy through interactions with others who understand through their own lived experience^19,24^. Although there is some evidence that informal carers of persons with PD may benefit from peer-to-peer support programs^25,26^, there is little consensus on how such programs should be delivered to optimally meet the needs of the informal carers.

During the care process, informal carers of persons with PD gain unique knowledge from their own practical experiences in dealing with the challenges of PD care. Yet, this relevant and unique knowledge often remains unused, and in fact disappears entirely once the loved one with PD has passed away. This is really a missed opportunity: former carers could share their experiences and offer information as well as advice to prepare and support those who are currently active as carers. Instead of involving fellow carers who currently also care for persons with PD themselves, former carers could take the role of “peers” and offer support to current carers. A mixed methods study revealed that current carers of people with Alzheimer’s disease benefit from such a peer-to-peer support^27^. Importantly, the former carers also perceived this role as gratifying, because it was emotionally rewarding to share one’s own experiences with others^27^. Indeed, previous research with former carers of people with Alzheimer’s disease as peers has shown positive effects – better coping with loss of the loved one, increased self-confidence by sharing coping strategies, and declined feelings of loneliness^24^. However, there is only little research available that evaluated the additional value of incorporating former informal carers into peer-to-peer support programs, and those studies that are available deal with other diseases than PD. Therefore, we aimed to examine the support needs and willingness of both current and former carers of people with PD to share experiences as peers.

## Results

### Participant characteristics

Characteristics of the participating current and former informal carers are reported in Table 1. In total, 141 current carers and 15 former carers completed the questionnaire. Most informal current carers were women (68%), mainly living as partner in the same household with the person with PD (87%). The average age of the current carers was 68 years. Most carers had provided informal care between one and eight years. Most carers cared for the person with PD day and night (45%) or during the day (28%). About one-third of the current carers reported to no burden or being hardly burned. A minority of 12% reported to feel heavily burdened (9%) or overburdened (3%).

**Table 1:** Study sample characteristics.

	Caregivers (*N* = 141)	Former caregivers (*N* = 15)^a^
Caregiver characteristics		
Gender (%)		
Women	96 (68.1)	12 (80.0)
Age in years, mean ± SD	68.0 ± 7.8	67.5 ± 10.7
Relationship to patient (%)		
Spouse	134 (95.0)	8 (53.3)
Mother/father	4 (2.8)	7 (46.7)
Grandmother/grandfather	–	–
Daughter/son	–	–
Sister/brother	2 (1.4)	–
Friend/acquaintance/colleague	1 (0.7)	–
Patient characteristics		
Gender (%)		
Women	49 (34.8)	7 (46.7)
Age in years (mean ± SD)	70.6 ± 6.5	–
Living situation (%)		
Living alone	5 (3.5)	1 (6.7)
Living in a household with partner	122 (86.5)	9 (60.0)
Live in a household with partner and children	5 (3.5)	–
Staying in the household of the children	2 (1.4)	–
Living in a facility (i.e. nursing or residential home)	4 (2.8)	1 (6.7)
Other	3 (2.1)	4 (26.7)
First symptoms of PD (%)		
Less than a year	1 (0.7)	n/a
1–5 years	45 (31.9)	n/a
5–10 years	47 (33.4)	n/a
10–15 years	24 (17.0)	n/a
More than 15 years	24 (17.0)	n/a
Death of person with PD (%) (*N* = 14)		
Less than a year	n/a	5 (33.3)
1– 3 years ago	n/a	2 (13.3)
3–5 years ago	n/a	2 (13.3)
5 years or longer	n/a	5 (33.3)
Care taking		
Frequency (%)		
Day and night	63 (44.7)	9 (60.0)
During the day	40 (28.4)	3 (20.0)
3–6 times per week	6 (4.3)	1 (6.7)
1–2 times per week	4 (2.8)	–
Less than once per week	7 (5.0)	–
Less than once per month	5 (3.5)	–
Very variable	16 (11.3)	2 (13.3)
Years of care taking (%)		
Less than 6 months	10 (7.1)	–
Between 6 months and a year	7 (5.0)	1 (6.7)
1–2 years	20 (14.2)	–
2–3 years	21 (14.9)	1 (6.7)
3–5 years	16 (11.3)	2 (13.3)
5–8 years	24 (17.0)	1 (6.7)
8–10 years	14 (9.9)	1 (6.7)
More than 10 years	29 (20.6)	9 (60.0)
Caregiver burden		
Perseverance time (%), *N* = 140		
Less than a week	–	n/a
More than 1 week, less than 1 month	1 (0.7)	n/a
More than 1 month, less than 6 months	4 (2.8)	n/a
More than 6 months, less than 1 year	8 (5.7)	n/a
More than 1 year, less than 2 years	8 (5.7)	n/a
More than 2 years	119 (84.4)	n/a
Perceived caregiver burden (%)		
Not or hardly burdened	44 (31.2)	n/a
Somewhat burdened	48 (34.0)	n/a
Rather heavily burdened	33 (23.4)	n/a
Heavily burdened	12 (8.5)	n/a
Overburdened	4 (2.8)	n/a

*^a^one former caregiver is still caring for a person with PD who is living in a residential home. Therefore she considers herself a former caregiver; *n/a* not applicable.

Regarding former carers, most were women (80%) and either a partner (53%) or child (47%), predominantly living in the same household with the person with PD. The average age of the former carers was 67 years. 60% had been providing informal care for more than 10 years, 27% less than 5 years and 13% more than 5 but less than 10 years. The majority provided care during day and night (60%), and 20% during the day. One third of former carers reported that they lost the person they cared for in recent years, and one third lost their partner five years ago or longer.

### Unmet support needs

The majority of both current and former informal carers received support while taking care for a person with PD. The support was not only provided by healthcare professionals such as neurologists, PD nurses and physiotherapists, but also through their own social network, including family members, friends and neighbors (Table 2). Current carers were asked whether they wanted to receive additional support: 45% gave a positive answer to this question, 35% said that they did not need any additional support, and 20% were unsure whether they wanted to receive any additional support. In addition, we found that the majority of those current carers that gave a positive answer also reported that they felt more burdened: 55.6% felt heavily burdened or overburdened. By contrast, 86% of those who gave a negative answer felt not or somewhat burdened. A re-emerging reason for additional support was that current carers felt they were on their own, while feeling a need for information and practical advice (“*it is a search for the right information and tools. So, often I have the feeling that I need to re-invent the wheel by myself”)*, seeking recognition for their role as informal carers by being heard *(“It is not about the support, but about being seen and heard. The focus is on my partner – I often have to take action in order to be heard as well in a conversation”)*, and wanting to know how to care for themselves (“*How do I combine care with my own life, which sometimes turns out to be very difficult due to unpredictability“*).

**Table 2:** Support resources and support needs.

	Caregivers (*N* = 141)	Former caregivers (*N* = 15)
Received support (%)		
I received support/advice via newsletter or websites (i.e., Parkinson Association, ParkinsonNet)	54 (38.3)	5 (33.3)
I received support/advice from my direct environment (i.e., family, friends, neighbors	44 (31.2)	8 (53.3)
I received support/advice via book(s)	32 (22.7)	4 (26.7)
I did not (yet) receive support or advice	43 (30.5)	3 (20.2)
I received support/advice from other informal caregivers during a group meeting (i.e. Parkinson Café)	22 (15.6)	5 (33.3)
I received support/advice from other informal caregivers, through individual contact (i.e., in personal-contact, online community, forum)	12 (8.5)	1 (6.7)
Topics for additional support (%)	70 (49.6)	n/a
Finding balance between caring for the person with PD and caring for myself	62 (44.0)	n/a
How I as an informal caregiver can recognized signals (e.g. depression, dementia, hallucinations) in the person I care for	59 (41.8)	n/a
Learning to communicate based on your own needs and wishes	54 (38.3)	n/a
Dealing with psychosocial consequences (e.g., anxiety, depression) in PD	37 (26.2)	n/a
Dealing with a changing relationship with the person with PD	36 (25.5)	n/a
Taking care of myself as a caregiver	35 (24.8)	n/a
Information about how to receive social and/or financial support	33 (23.4)	n/a
Developing skills for dealing with stress (situations)	28 (19.9)	n/a
Information about PD and treatment	28 (19.9)	n/a
Dealing with changing relations and reactions from the environment	27 (19.1)	n/a
Information about possible strategies for dealing with stress	25 (17.7)	n/a
Accepting my own/new role as an informal caregiver	21 (14.9)	n/a
Other	30 (21.3)	n/a
Willing to share experience as peers (%)	71 (50.4)	13 (86.7)

Those topics reemerged when asked to identify the most important topics for additional support: ‘finding a balance between caring for my loved one and caring for myself’ (50%), ‘dealing with a changing relationship with the person with PD’ (44%), ‘how to recognize signals and symptoms of PD (e.g., depression, hallucinations) as carers (42%), and ‘dealing with a lack of emotion, motivation or enthusiasm and how to stimulate my loved one’ (38%).

### Sharing experiences as peers

Slightly more than half of the participating current informal carers indicated that they would like to get into contact with former informal carers to share their experiences and knowledge with them (Table 2). From those who indicate to want receive additional support, 62% indicated to would like to share experiences with former carers. In the open questions, several respondents emphasized that former informal carers could provide information and practical advice on the caring process (“*the future is like a black box for me and I find hardly any information about it”*). Other frequently mentioned reasons were to feel recognized as *carers* and to receive emotional support (“*I think recognition is very important and former carers have gone through that whole process”; “I want to talk freely with someone who knows my situation. I would hope for some advice, for understanding, for a restful arm around me, for someone to tell me that I cannot do more than my best”)*. The other half of participating current informal carers reported to have no interest to contact former informal carers. Main underlying reasons were that they had other support resources and were currently not in a situation where they needed additional information (“*I’m not that far in the process yet”; “I solve everything with my partner. If not, I ask the nurse, physiotherapist, general practitioner or I search on the internet”)*. Other given reasons were being afraid with the future perspective (“*I want to avoid the future perspective as much as possible. The now is more important*”) and having enough information and important contacts with others (“*Because I have enough people around me who think along and assist me*”, “*The information that we receive from our neurologist and the Parkinson’s nurse is for now sufficient for us*”).

A large majority (13/15) of participating former informal carers also indicated a willingness to share their experiences and knowledge with current carers. In the open question, they reported that they wanted to help and support those who are now providing care to a person with PD (“*Because I believe that I have valuable experience to provide carers as well as the person they care for the opportunity to make choices that they otherwise would avoid and which they might regret in a later sta*ge”). Two former informal carers reported that they did not want to share experiences and knowledge with current carers, because of the recent loss of the person with PD (less than a year).

In addition, former informal carers indicated that the main underlying motivational reason for peer support was that current informal carers could benefit from shared experiences (92%), followed by the motivation to use their own knowledge and skills to support current carers (69%) and their experiences of a lack of support when they were providing informal care themselves (30%) (see Table 3).

**Table 3:** Motivational reasons of former caregivers for sharing experiences with current caregivers (*N* = 13).

I want to share my experiences so that other caregivers can benefit from it	12 (92.3)
I want to use my knowledge and skills	9 (69.2)
I experienced a lack of support myself when I was an informal caregiver	4 (30.8)
I would like to give something back to other caregivers	3 (23.1)
I would like work as a volunteer^	2 (15.4)
I am looking for a (new) challenge	1 (7.7)
I would like work as a volunteer	1 (7.7)
I would like to meet other people	1 (7.7)
I feel morally obligated	1 (7.7)

### Design preferences

Table 4 presents the results of the questionnaire dealing with design wishes for a peer-to-peer support initiative, which would enable current and former informal carers to share experiences and knowledge with each other. Regarding the setting, participants in both groups (percentages for current and former informal carers respectively) preferred to drink coffee together (78%; 61%), have a walk together (75%; 46%), to organize support group meetings with informal carers (68%; 77%), or a lecture or workshop led by informal carers (76%; 46%).

**Table 4:** Setting, form and conditions for a peer-to-peer intervention connecting current and former informal caregivers.

	Caregivers (*N* = 71)*	Former caregivers (*N* = 13)*
Kind of support (%)^a^		
Practical support	66 (93.0)	8 (61.5)
Exchange experience with former caregivers	61 (85.9)	5 (38.5)
Someone who will listen to my story	55 (77.5)	11 (84.6)
Advice and guidance	47 (66.2)	7 (53.8)
Emotional support	43 (60.6)	6 (46.2)
Other	7 (9.9)	4 (30.8)
Type of contact (%)		
Drinking a cup of coffee together in a café	55 (77.5)	8 (61.5)
Lecture or workshop led by former informal caregivers	54 (76.1)	7 (46.2)
During a walk	53 (74.6)	6 (46.2)
Overview of former informal caregivers living nearby	51 (71.8)	6 (46.2)
Group meeting with changing topics	48 (67.6)	10 (76.9)
Support group meeting with former informal caregivers	48 (67.6)	9 (69.2)
Consultation hour with former informal caregivers to discuss practical problems	37 (52.1)	8 (61.5)
Via a manual entailing advices and tips	34 (47.9)	7 (53.8)
Online forum or private group of (former) informal caregivers	32 (45.1)	5 (38.5)
Via online contact (mails or WhatsApp)	31 (43.7)	7 (53.8)
Conversation at your home	30 (42.3)	8 (61.5)
Via telephone contact	28 (39.4)	6 (46.2)
Via a theater play about experiences and advice	26 (36.6)	5 (38.5)
Blog of a former informal caregiver with the opportunity to respond from one’s own situation	24 (33.8)	3 (23.1)
Via video contact (i.e., Skype, Zoom, FaceTime)	17 (23.9)	7 (53.8)
Design preferences (%)		
In-person vs online		
In-person contact	30 (42.3)	6 (46.2)
Online contact	8 (11.3)	–
Combination of in-person and online contact	33 (46.5)	7 (53.8)
Individual vs group meeting		
Individual contact	18 (25.4)	3 (23.1)
Group meeting	14 (19.7)	2 (14.4)
Combination of individual and group meeting	39 (54.9)	8 (61.5)
Contact frequency		
Daily	–	–
1–2 times a week	–	2 (15.4)
Less than once a week	9 (12.7)	1 (7.7)
Less than once a month	13 (18.3)	5 (38.5)
Variable, depending on when I/the caregiver need(s) it	49 (69.0)	5 (38.5)
Day of the week		
During the week	26 (36.6)	3 (23.1)
At the weekend	1 (1.4)	–
No preference	44 (62.0)	10 (79.9)
Duration		
One-time contact	–	1 (7.7)
Several times	1 (1.4)	5 (38.5)
Long-term contact	2 (2.8)	–
Not to say in advance, as long as I need it	68 (95.8)	7 (53.8)
Spending time per week		
1–2 h	59 (83.1)	8 (61.5)
One half day per week	11 (15.5)	3 (23.1)
One day a week	–	1 (7.7)
Several days a week	1 (1.4)	1 (7.7)
Condition: I think it is most important that the (formal) caregiver (%)^b^		
Has a degree of baseline compatibility	54 (76.1)	6 (46.2)
Is reliable	44 (62.0)	n/a
Has made comparable experiences as a caregiver	42 (59.2)	5 (38.5)
Is a good listener	42 (59.2)	n/a
Can communicate well	42 (59.2)	n/a
Can empathize with my situation	36 (50.7)	n/a
Is a regular person	29 (40.8)	n/a
Has gained lots of experience as a caregiver	25 (35.2)	n/a
Has the same background (age, relationship to person with PD)	25 (35.2)	–
Lives nearby	22 (31.0)	2 (15.4)
Can handle emotions well	16 (22.5)	n/a
Has the same interests (being like-minded)	14 (19.7)	2 (15.4)
Is patient	11 (15.5)	n/a
Does not live nearby	1 (1.4)	–
Is always available	–	n/a
Has gained little experience until now	n/a	4 (30.8)

^a^Only those filled in the questions that were willing to share their experience with current and former caregivers, ^b^Multiple answers possible, *n/a* Not applicable.

Regarding formal aspects, most participants in both groups preferred individual contact, or a combination of individual and group meetings. Contact could either be in-person, or a combination of in-person and online meetings. According to the answers given, contact frequency should be based on the current and former informal carers need for contact. No preferences were mentioned regarding day of the week and duration of the contact. In both groups, the majority reported to be able to spend 1–2 h per week on the peer-to-peer contact.

In both groups, having a baseline commonality with the peer was mentioned as an essential requirement for successful peer-to-peer support. Current carers also reported that they found it important that the former carers is reliable, can empathize with the situation, and can listen and communicate well. Over one-third of former informal carers reported that they found it important that the current informal carers has comparable experiences with caring for a person with PD.

## Discussion

This study shows that half of the current informal carers expressed a need for additional support in caring for a person with PD. In addition, our findings indicate the potential of former informal carers as peers to enhance support for informal carers who are currently taking care of a loved one with PD, by sharing their own experiences and knowledge.

Given the expected increase in the number of people with PD, together with a limited availability of health care professionals specialized in PD care^28^, informal carers will become increasingly important for ensuring the continuity of PD care. Identifying informal carers support needs – in addition to the information, advice and support they receive from medical specialists and health care professionals – provides important directions for the development of peer support initiatives that could benefit and support informal carers. With this study, we aimed to identify the needs for additional support among informal carers of people with PD, to map the desire to share knowledge and experiences between current and former informal carers, and to design the contours of peer-to-peer support initiatives.

Our results indicate that most current informal carers already received support, among others from allied health professionals and their own social environment. Yet, a considerable percentage (45%) expressed a need for additional support, especially in the form of information provision, a listening ear, emotional support and recognition in their role as carers. This is in line with previous research among a smaller group of 26 informal family carers of people with PD, which revealed an information deficit and need for social support among informal carers^18^. Moreover, peer-to-peer support groups for PD have positive effects on informal carers. Specifically, these peer groups provide a secure place to exchange information and experiences, receive emotional support and advocacy, enhanced self-confidence and reduced feeling of loneliness^19,21^. Our study focuses on the potential of former informal carers as peers. They can relate from their lived experience and address the information deficit, offer emotional support and meet the needs and wishes of current informal carers. In addition, contact with former informal carers could also facilitate social networks, which are important factors for preventing strains of the carers role and improving quality of life of carers^29,30^. However, informal carers often experience barriers in sustaining social activities and diminished social contacts, leading to less emotional support and a feeling of loss and loneliness^21,31^.

Regarding setting and content, this study showed that current and former carers preferred to meet each other personally over online contact and preferred individual contact or a combination of individual contact and group meetings. Our results further showed that mere online contact was less appealing for both groups. However, in case of circumstances that do not allow for in-personal contact – e.g., during the social distancing regulations during the COVID-19 pandemic, or for carers unable to leave the person with PD alone at home – online communities might be a well-suited alternative. Previous research showed that informal carers of people with dementia reported beneficial effects of participating in online communities^32^, however, little research has been done on integrating former informal carers as moderators of such online communities.

This study was not without shortcomings. Although we aimed to include a diverse group of participants by formulating only limited in- and exclusion criteria, some groups were underrepresented in our study. First, we primarily included mildly burdened informal carers which can also be derived from the average short duration of being a carer. As the disease progresses, the burden of caring for a person with PD increases and might this be accompanied by a change in support needs. It follows that carers who were moderately or even highly burdened and their (peer-) support needs were possibly underrepresented. Moreover, it would be interesting to include questions on the severity of motor and non-motor symptoms as it is reasonable that specific symptoms, for example, cognitive decline, apathy or depression, have a distinct impact on carers burden and might influence specific carers need to seek for emotional, instrumental and informational support from others experiencing similar situations. Second, most carers were partners, while the average age of participants was high – only few younger (age < 50 years) carers participated. Hence, we did not reach all populations, including children who are taking the role as carers and those who are caring for a person with Young Onset PD (YOPD). Those groups might deal with different stressors and support needs, e.g. financial situation, perceived restrictions on daily and social activities, and finding balance between work activities or raising children and one’s role as a carer. Our results likely underestimate the support needs of those specific groups. Therefore, future studies should focus on finding ways to include those group that are more difficult to reach. For instance, high burned caregivers might be reached through active involvement of health care professionals or home visits. Third, only a small group of former informal carers participated. Recruitment of former informal carers proved challenging as they often disappear out of sight after the person with PD has passed away. It follows that new recruitment strategies are needed that allow to stay in contact with carers after the loved one has died. For instance, a signed contact form which allows to approach the former carers. Additionally it would be helpful to also explore together with former carers about the most appropriate time and way to be contacted for requests and how to stay in touch with those willing to be a peer, but who do not feel that it is the right time point (e.g. still coping with bereavement). Fourth, the current study only focused on carers living in the Netherlands. Cultural values and perspectives, including family caregiving, privacy behavior and gaining rewards from caregiving, are likely to impact the perceived burden and support needs of carers. Therefore, a cross-cultural study design would be needed to expand our knowledge on (peer) support needs across different countries.

Moreover, our findings might be biased, because the current and former informal carers that did volunteer might be the ones that are most enthusiastic about the idea of sharing their experiences and knowledge. Indeed, several of these informal carers were still actively involved in PD-related organizations, such as the Dutch Parkinson’s Disease Association, and enjoyed these activities. But importantly, even though we cannot offer an accurate estimate of the overall willingness of formal carers to act as peer supporters, our findings do offer a clear signal that there is potential to develop such a peer-to-peer program, with active involvement of former carers. Finally, our recruitment was restricted by the current COVID-19 pandemic which only permitted online recruitment through newsletters and websites, whereas flyers, oral presentations and in-person contact might have led to more study enrolments, especially among carers that are not so familiar with technology.

However, while dependent on the cultural context and study sample, our findings should serves as a source of inspiration to stimulate further research on the topic from which more robust conclusion can be drawn and new innovations that support carers can be developed.

## Methods

We conducted a cross-sectional questionnaire study among current and former carers of persons with PD in the Netherlands. Participants were enrolled from August till December 2020. Current and former carers of persons with PD, able to complete the questionnaire in Dutch, and willing to participate were eligible for this study.

Recruitment took place through different channels. First, the Dutch Parkinson’s Disease Association posted a recruitment announcement on their website and in their newsletter. In total, 80 informal carers responded on the recruitment announcement and of these, 58 (73%) completed the questionnaires. Second, invitation letters were sent to members of ParkinsonNext, a platform for patients with PD and their carers interested in participating in research. 75 current or former informal carers reacted to the recruitment announcement, of whom 40 (53%) completed the questionnaire. Third, carers participating in the PRIME Parkinson Evaluation study^33^ (a prospective observational study among people with parkinsonism, informal carers and healthcare professionals in the Netherlands) were asked to participate in additional studies regarding carers burden. In total, 289 of 564 participating current informal carers in the PRIME study responded positively. Of these, 60 informal carers were interested to participate in the present study and 53 (88%) completed the questionnaires. 27 responded on the invite to not feel eligible for the study as they provide no or little care for a person with PD. Finally, calls for participation were made through social media channels of the authors which resulted in successful recruitment of two former informal carers who both participated.

Data were collected using a self-administered electronic questionnaire, or if requested, through a paper-based questionnaire. For the purpose of this study, two versions of the questionnaire were developed: one for former carers and one for current carers of persons with PD. The questionnaires were designed based on a former study among carers of persons with dementia^34^, a literature study (unpublished data) and through discussions with a patient researcher, a current carer and a former carer.

Both questionnaires were divided into four parts, with different topics. The first part identified currently used resources for support, as well as unmet support needs of current and former carers, through close-ended questions. The second part consisted of closed-ended questions to investigate on which topics current informal carers wanted to receive support or practical advice. In the third part, a combination of closed and open-ended questions was used to examine wishes and needs for a new peer-to-peer support initiative connecting current and former carers, including questions about content, location, length of contact and type of contact (i.e., individual vs group, physical vs digital). Finally, a set of closed-ended questions were used to capture the respondent’s socio-demographic characteristics as well as demographics of the person with PD they currently care or have cared for. Moreover, the questionnaire for current carers included a question on perseverance time^35^ and one question on subjective carers burden. Questionnaires were pre-tested within a sample of six current and one former carers and were improved accordingly to ensure readability, clearness and completeness of the questions. The results of the questionnaire study were discussed with an expert group consisting of one patient, four current carers and two former carers with the aim to discuss the implications of those results and formulate recommendations for future peer-to-peer support initiatives.

Questionnaire data were inserted in Castor EDC, either directly by respondents completing the electronic version of the questionnaire or, in case of paper-based versions, manually by a researcher [ADvH]. All statistical analyses were performed using SPSS 25 statistic software. Continuous variables were summarized using mean and standard deviation (SD) and categorical variables were expressed as absolute (number) and relative frequencies (percentage). Open questions were analyzed thematically, after open coding by one author [ADvH] and discussed with a second author [MvdM]. These data were transformed into percentages and used to illustrate current and former carers opinions.

### Ethics

The study protocol has been reviewed by the Ethical Board of the Radboud university medical center (file number 2020–6304), and it was deemed exempt from further ethical approval. All participants received an information letter and provided written informed consent before study inclusion.

## Data Availability

The datasets generated during the present study are available on request from the corresponding author.
